# Study on Lubrication Characteristics of Metal Liquid Film Based on Electromagnetic-Elastic Mechanics-Hydrodynamics Multiphysics Coupling Model

**DOI:** 10.3390/ma13051056

**Published:** 2020-02-27

**Authors:** Chengxian Li, Shengguo Xia

**Affiliations:** 1 Key Laboratory of Pulsed Power Technology (Huazhong University of Science and Technology), Ministry of Education, Wuhan 430074, China; axian22ruru@163.com; 2 State Key Laboratory of Advanced Electromagnetic Engineering and Technology (School of Electrical and Electronic Engineering, Huazhong University of Science and Technology), Wuhan 430074, China

**Keywords:** metal liquid film, magnetic pressure, fluid pressure, film thickness, transition, lubrication effect

## Abstract

In an electromagnetic rail launcher, a metal liquid film is created at the armature/rail (A/R) contact interface. It has a significant impact on electromagnetic launch performance. In this paper, an electromagnetic-elastic mechanics-hydrodynamics multi physics coupling model is established in consideration of the metal liquid film’s own acceleration, magnetic pressure and dynamic changes in film thickness. Based on this model, the lubricating characteristics of magnetic pressure and fluid pressure distribution, film thickness distribution and velocity distribution of the metal liquid film were studied. When the velocity of the metal liquid film is very fast, and the magnetic pressure is reduced, it may fail to maintain stability and rupture, which may be an important reason for the transition. Finally, this paper analyzes the lubrication effect of the metal liquid film, and points out that when we want strictly to control the muzzle velocity, the lubrication effect of the metal liquid film must be considered.

## 1. Introduction

Electromagnetic launcher can convert the electromagnetic energy provided by the high-power pulse power source into the kinetic energy of the payload, and utilize the electromagnetic force to drive the projectile to accelerate between two rails, therefore the projectile can obtain an ultra-high velocity of 2500 m/s or more. However, a large amount of Joule heat is generated due to the MA-level current. Coupled with the frictional heat generated during the sliding process, the armature surface will be melted, and a metal liquid film will be generated between the armature and the rail. It will be deposited on the surface of the rail after launch [1,2,3,4]. Especially under certain armature geometries, the current distribution at the A/R interface are very uneven, which is more likely to cause the surface of the armature’s tail to melt [5,6,7,8,9]. After the surface of armature’s tail are melted, it may lead to transition, however, on the other hand, as well as reducing the friction and improving the electrical conductivity of the A/R contact area [10,11,12]. Therefore, it is important to study the influence of the armature melting and lubrication characteristics of metal liquid film on launch performance.

Stiffer first established a hydrodynamic model of the metal liquid film, and obtained analytical expressions of the film thickness, friction coefficient, and wear rate of the liquid film in a quasi-steady state. However, their model assumption is too simple. They consider the shape of the liquid film remains unchanged. Furthermore, the pressure change in the liquid film is not considered in the Reynolds equation [13]. Stefani coupled fluid dynamics and multi-phase heat transfer, and established a thermodynamic model of the metal liquid film. Based on this model, the turbulence effect on the model was considered. However, the calculation results and experimental results are quite different [14,15]. V. S. Yuferev’s team studied the electromagnetic, force, and thermal characteristics of the metal liquid film while ignoring the dynamic changes in the thickness of the liquid film and defining the armature shape as a rectangle [16,17]. Thiagarajan and Heish established a two-dimensional magnetohydrodynamic model that considers the effects of electromagnetic forces, however they also ignored the effect of dynamic changes in the film thickness on the characteristics of the metal liquid film [18]. A thermodynamic-magnetohydrodynamic model for the metal liquid film at the A/R interface with external lubricant injection was established by Wang Lei to study the effect of the liquid film on the characteristics of the A/R system; however, the actual source of the liquid film is the armature itself. The self-lubricating model is more realistic than Wang Lei’s external lubrication model [19,20]. At the same time, the above model does not consider the influence of the liquid film’s own acceleration on the film thickness, fluid pressure distribution and velocity distribution. While the metal liquid film is conducting, it is also subject to lorentz force, then it must have its own acceleration. In studying the properties of the metal liquid film, Yao Jinming considered the effects of the metal liquid film’s own acceleration and magnetic pressure; however, she ignored the effect of the dynamic changes in film thickness on the characteristics of the liquid film [21,22].

In this paper, we consider the dynamic changes of film thickness, the effects of the liquid film’s own acceleration and magnetic pressure, and then establish an electromagnetic-elastic mechanics-hydrodynamics multiphysics coupling model. We then use this model to analyze the dynamic changes of the magnetic pressure and fluid pressure distribution, film thickness distribution and velocity distribution of the liquid film during the launch process. Finally, this paper studies the lubrication effect of metal liquid film.

## 2. Electromagnetic-Elastic Mechanics-Hydrodynamics Multiphysics Coupling Model

**Figure 1:**
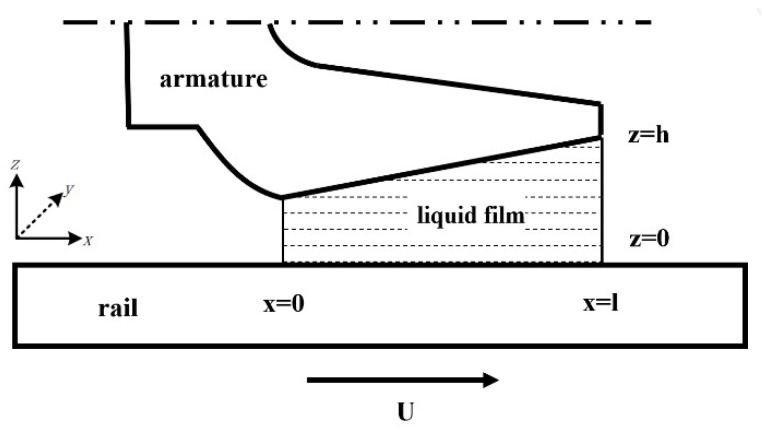
Schematic diagram of the liquid film of the A/R contact interface.

The electromagnetic railgun works under the extreme conditions of high current and high velocity. Due to the continuous action of Joule heat and frictional heat, the armature surface material will continue to melt, and it will form a complete liquid film at the A/R contact interface. As shown in Figure 1, the x direction represents the direction of motion, the z direction is perpendicular to the rail direction, and the y direction is the armature width direction. In the calculation process, the armature is defined as the inertial reference frame; that is, the armature movement velocity is assumed to be 0, and the velocity of rail is ***U***.

In the electromagnetic launch process, the average thickness of the metal liquid film deposited on rail is about 50 μm, which is much larger than the maximum peak-to-valley distance (4.7 μm) corresponding to the rail and armature machining accuracy at the N8 level. Therefore, the metal liquid film will be completely filled between the peaks and troughs of the surface roughness, and its typical thickness is much larger than five times the roughness. According to the tribological theory, the lubrication state of the metal liquid film is in a hydrodynamic lubrication state. If the role of the elastic deformation of the armature as a whole in hydrodynamic lubrication is considered, the lubrication state of the metal liquid film will be in the hydrodynamics lubrication, and both states can be studied by the Reynolds equation. However, the solution to the Reynolds equation requires the film thickness of the metal liquid film as the initial condition, and the film thickness is obtained by solving the displacement equation of the armature subjected to the electromagnetic force and the fluid pressure of the metal liquid film. Therefore, the entire calculation model includes three parts: electromagnetic module, solid mechanics module and fluid dynamics module.

### 2.1. Fluid Dynamics Module of Metal Liquid Film

**Figure 2:**
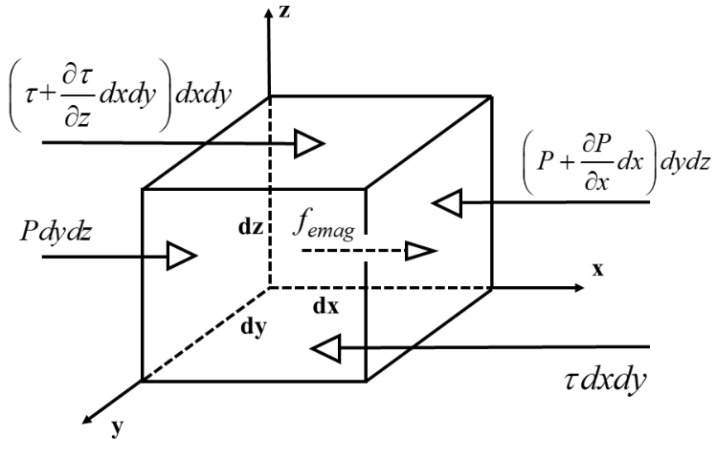
The force analysis of the liquid film infinitesimal element.

When the armature accelerates, the metal liquid film at the A/R interface is subjected to inertial force, at the same time the current flows through the metal liquid film, therefore it is subjected to electromagnetic force. The melted wear on the armature surface directly determines the shape distribution of the liquid film. Therefore, these factors must be considered comprehensively when deriving the Reynolds equation. The force analysis of the liquid film infinitesimal element is shown in Figure 2.

According to ΣF=ma, we have,

(1) $$\begin{array}{l} (Pdydz+(\tau +\frac{\partial \tau }{\partial z}dz)dxdy+f_{emag}dxdydz)-((P+\frac{\partial P}{\partial x}dx)dydz+\tau dxdy)+\rho a_{a}dxdydz\\ =\rho a_{f}dxdydz \end{array}$$

After simplification, we can get equation of,

(2) $$\frac{\partial \tau }{\partial z}+f_{emag}+\rho a_{a}-\frac{\partial P}{\partial x}=\rho a_{f}$$

where, P is the fluid pressure, τ is the shear force that the liquid film infinitesimal element receives in the x-axis direction, $f_{emag}$ is the lorentz forces on the liquid film in the longitudinal direction, which allows to be represented by a magnetic pressure, ρ is density of liquid film, $a_{a}$ is the acceleration of the armature, $a_{f}$ is the self-acceleration of the film.

The shear force and electromagnetic force calculation formulas of the infinitesimal element are as follows:

(3) $$\tau =\eta \frac{\partial u}{\partial z}$$

(4) $$f_{emag}=-\frac{1}{2\mu_{0}}\frac{\partial B_{}^{2}}{\partial x}=-\frac{\partial P_{M}}{\partial x}$$

where, $P_{M}$ is magnetic pressure, we specify the total pressure $P_{\Sigma }$ is the sum of the fluid pressure P and the magnetic pressure $P_{M}$, then we have,

(5) $$\eta \frac{\partial^{2}u}{\partial^{2}z}=\frac{\partial P_{\Sigma }}{\partial x}-\rho (a_{a}-a_{f})$$

Due to the liquid film being very thin, it can be assumed that the pressure remains constant in the thickness direction. According to Equation (5), by integrating along the thickness direction of the liquid film, we can get the velocity of the liquid film.

(6) $$u=\frac{1}{2\eta }\left(\frac{\partial P_{\Sigma }}{\partial x}-\rho (a_{a}-a_{f})\right)(z^{2}-zh)+U\left(1-\frac{z}{h}\right)$$

According to the no-slip boundary condition,

$$\left\{ \begin{array}{l} z=0,u=U\\ z=h,u=0 \end{array}\right.$$

integrate u along z direction, the flow rate $q_{x}$ of the liquefied layer in the x-direction unit width can be obtained:

(7) $$q_{x}=\int_{0}^{h}udz=\frac{-h^{3}}{12\eta }\left(\frac{\partial P_{\Sigma }}{\partial x}-\rho (a_{a}-a_{f})\right)+\frac{Uh}{2}$$

The fluid continuity equation is:

(8) $$\int_{0}^{h}\frac{\partial \rho u}{\partial x}dz+\int_{0}^{h}\frac{\partial \rho v}{\partial y}dz+\int_{0}^{h}\frac{\partial \rho w}{\partial z}dz+\int_{0}^{h}\frac{\partial \rho }{\partial t}dz=0$$

Assuming that the liquid film velocity does not change in the y direction, regardless of the variable density effect, then:

(9) $$\frac{\partial }{\partial x}q_{x}-v_{m}=0$$

where $v_{m}$ is defined as the outflow velocity of the liquid film from the A/R contact interface along the z direction, its value is equal to the melt wear rate of the armature surface.

Simultaneous Equations (7) and (9) can get the Reynolds equation expression as follows:

(10) $$\frac{\partial }{\partial x}(\frac{h^{3}}{12\eta }\cdot \frac{\partial P_{\Sigma }}{\partial x})=\left(\frac{U}{2}+\frac{\rho \cdot (a_{a}-a_{f})\cdot h^{2}}{4\eta }\right)\cdot \frac{\partial h}{\partial x}-v_{m}$$

The boundary condition for solving the Reynolds equation is

(11) $$\left\{ \begin{array}{l} x=0,P_{\Sigma }=P_{0}\\ x=l,P_{\Sigma }=P_{0}+P_{M} \end{array}\right.$$

where, $P_{0}$ is atmospheric pressure, l is the length of the liquid film, $P_{M}$ is the magnetic pressure at the outlet of the liquid film, and its magnitude is calculated by the following formula [10]:

(12) $$P_{M}=\frac{L’\cdot I^{2}}{2S}$$

where, $L^{\prime }$, I and S represent the inductance gradient, value of driving current and cross-sectional area of the launching system, respectively. It can be seen from the boundary conditions that the solution to the Reynolds equation first requires the solution to obtain the magnetic field distribution of the whole system, which is given in Section 2.2.

### 2.2. Electromagnetic Field Calculation Model

The physical size of the electromagnetic launch system is much smaller than the wavelength of the electromagnetic wave. At the same time, the skin effect and eddy current effect generated during the launch process will make the conduction current much larger than the displacement current; therefore, the influence of the displacement current can be ignored, and the entire launch system is regarded as a magnetic quasi-static field. The governing equation for calculating the electromagnetic field is as follows:

(13) $$\nabla \times B=\mu_{0}\cdot J$$

(14) $$\nabla \times E=-\frac{\partial B}{\partial t}$$

(15) J=σ⋅(E+U×B)

where ***B*** is the magnetic induction intensity, ***J*** is the current density, ***E*** is the electric field strength, *σ* is the conductivity of the conductor material, and *μ*_0_ is the magnetic permeability.

The magnetic diffusion equation of the whole system can be derived as follows:

(16) $$\frac{1}{\mu_{0}\sigma }(\nabla \times (\nabla \times B))+\frac{\partial B}{\partial t}=\nabla \times (U\times B)$$

The magnetic field distribution of the entire launch system is mainly concentrated on the y direction of Figure 1, and the magnetic fields in the x and z directions are negligible. For the two-dimensional model, assuming that the magnetic field is evenly distributed over the y direction, the magnetic induction can be written as $\vec{B}=B_{y}(x,z)\cdot {\vec{e}}_{y}$. Considering that the typical thickness of the liquid film is much smaller than the size of the armature and the rail, the influence of the liquid film on the magnetic field distribution of the interface at the A/R contact is negligible. Therefore, the magnetic diffusion equations of the orbit and armature are as follows:

(17) $$\frac{\partial B_{r}}{\partial t}+U\frac{\partial B_{r}}{\partial x}=\frac{\partial }{\partial x}(\frac{1}{\mu_{r}\sigma_{r}}\frac{\partial B_{r}}{\partial x})+\frac{\partial }{\partial z}(\frac{1}{\mu_{r}\sigma_{r}}\frac{\partial B_{r}}{\partial z})$$

(18) $$\frac{\partial B_{a}}{\partial t}=\frac{\partial }{\partial x}(\frac{1}{\mu_{a}\sigma_{a}}\frac{\partial B_{a}}{\partial x})+\frac{\partial }{\partial z}(\frac{1}{\mu_{a}\sigma_{a}}\frac{\partial B_{a}}{\partial z})$$

where, $B_{a}$, $\mu_{a}$ and $\sigma_{a}$ represent the magnetic induction, magnetic permeability and electrical conductivity of the armature, respectively, and $B_{r}$, $\mu_{r}$ and $\sigma_{r}$ represent the magnetic induction, magnetic permeability and electrical conductivity of the rail. At the A/R contact interface, the magnetic induction into the armature side and the rail side is equal to the normal direction. According to the above magnetic diffusion equation and boundary conditions, the magnetic field distribution of the armature and rails can be solved, and the current distribution and electromagnetic force distribution in the armature and orbit can be further solved.

(19) $$J_{x}=-\frac{1}{\mu_{0}}\frac{\partial B_{y}}{\partial z}$$

(20) $$J_{z}=\frac{1}{\mu_{0}}\frac{\partial B_{y}}{\partial x}$$

(21) $$f_{x}=-J_{z}B_{y}=-\frac{1}{\mu_{0}}B_{y}\frac{\partial B_{y}}{\partial x}$$

(22) $$f_{z}=J_{x}B_{y}=-\frac{1}{\mu_{0}}B_{y}\frac{\partial B_{y}}{\partial z}$$

where $J_{x}$ and $J_{z}$ are current density components in the x direction and the z direction, respectively, $f_{x}$ and $f_{z}$ are electromagnetic force components in the x direction and the z direction, respectively.

### 2.3. Calculation Model of Film Thickness Distribution of Metal Liquid Film

When the armature is deformed by the interaction of electromagnetic force and hydraulic pressure, a certain gap is formed into the A/R contact interfaces. At this point, the current has become very large, and the armature is also in a high-speed motion stage. This gap is completely filled by the metal liquid film produced by the armature melting.

**Figure 3:**
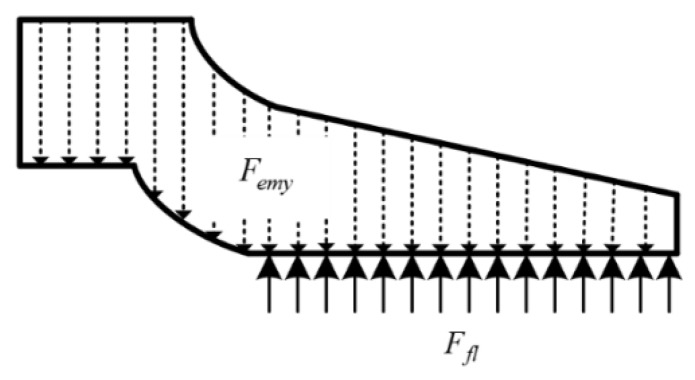
Force analysis of armature.

We assume that the shape variable of the armature is the thickness of the liquid film. Therefore, it is necessary to analyze the force of the armature and determine the amount of deformation. During the force analysis of the armature, as shown in Figure 3, the armature is subjected to two types of forces, namely electromagnetic pressure and fluid pressure. In the calculation process, the electromagnetic pressure is considered to be the bulk force acting on the entire armature, and the fluid pressure is considered to be the surface force acting only on the A/R contact interface.

Since the lower surface of the armature is limited by the rail, the armature is only likely to undergo an inward deformation. The entire deformation process can be performed using the elastic mechanics equations.

(23) ∇σ+F=0

(24) $$\sigma =\sigma_{0}+C_{\left(E,\nu \right)}(\varepsilon -\varepsilon_{0})$$

(25) $$\varepsilon =\frac{1}{2}(\nabla u+{\left(\nabla u\right)}^{T})$$

Equation (21) is a balance equation describing the relationship between stress and physical force when the armature is in equilibrium at a certain calculation time. Where σ is the stress tensor, and F is the volume force received by the armature, including electromagnetic pressure and fluid pressure. Equation (22) is an elastic constitutive equation that establishes the relationship between stress and strain, where ε is the strain tensor, which $\varepsilon_{0}$ is the initial strain tensor, which C is the fourth-order elastic sheet associated with Young’s modulus and Poisson’s ratio. $\sigma_{0}$ is the initial stress tensor; the formula (23) is the strain coordination equation, which ensures the integrity and continuity of the armature after deformation. In the formula, u is the displacement field to be determined, and the film thickness of the metal liquefaction layer is characterized.

### 2.4. Multiphysics Coupling Calculation Method

**Figure 4:**
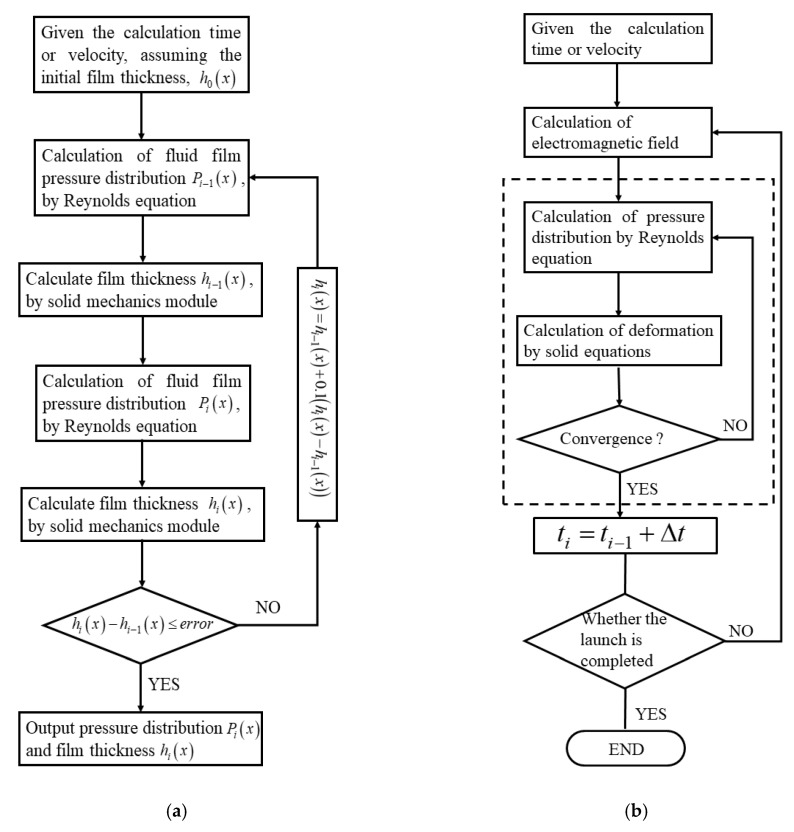
Calculation flow chart: (**a**) Iterative calculation flow chart, (**b**) Overall calculation flows chart.

The film thickness obtained by the elastic mechanics equation is only a single calculation result. Only the effect of liquid pressure on the film thickness of the liquid film is considered, and the influence of the film thickness of the liquid film on the liquid pressure distribution is not considered. In the actual calculation, the pressure and film thickness of the liquid film need to be iteratively calculated. The specific process is: given the calculation time and assuming an initial film thickness, the pressure distribution of the liquid film is calculated by the Reynolds equation, and then calculated the thickness of the liquid film by the solid mechanics module. Then, the film thickness of the metal liquid film is combined and converted into the Reynolds equation using a simple algebraic combination, and the obtained fluid pressure is combined with the electromagnetic pressure to generate a new film thickness. Repeat iteratively until the difference between the two calculated film thicknesses is less than 0.47 μm. The entire iterative process is shown in Figure 4a.

Figure 4a can only be calculated for a single moment or a specific A/R relative speed condition. Based on this, the entire current waveform is discretized, and the above research ideas are repeated using a cycle to obtain the metal liquid film during the entire launch process. The calculation flow of dynamic characteristic distribution, such as pressure and film thickness, is shown in Figure 4b. The part of the dashed box of Figure 4b is a simplified version of Figure 4a. The value of Δt is 0.001 ms.

**Figure 5:**
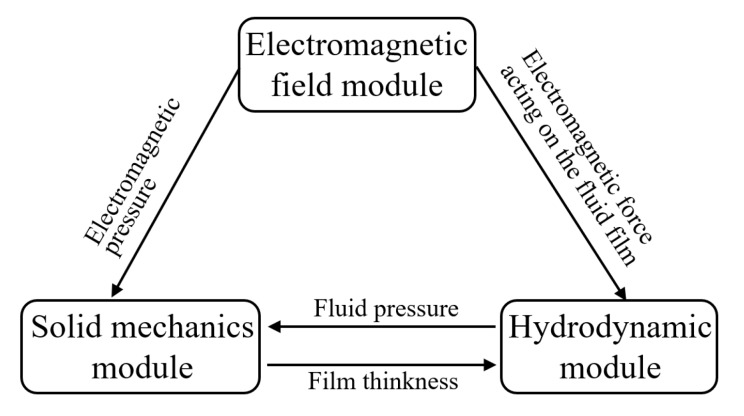
Schematic diagram of the coupled calculation of Electromagnetic-Elastic Mechanics-Hydrodynamics Multiphysics Coupling Model for metal liquid film.

The solution to the dynamic characteristics of the metal liquid film involves the calculation of coupling with multiple physical fields, including electromagnetic fields, fluid mechanics, and solid mechanics. The coupling relationship between them is shown in Figure 5. Solve the electromagnetic pressure in the z-direction of the armature tail using the electromagnetic module, and transfer it to the solid mechanics module to provide the component of the armature’s tail deformation. At the same time, solve the magnetic pressure in the metal liquid film and transfer it to the hydrodynamic module. Then, provide boundary conditions for solving the Reynolds equation. The coupling between the solid mechanics module and the hydrodynamic module is achieved through an iterative calculation between the pressure and the film thickness of the metal liquid film.

## 3. Calculation Conditions and Results Analysis

### 3.1. Calculation Conditions

The Electromagnetic-Elastic Mechanics-Hydrodynamics Multiphysics Coupling Model of the metal liquid film can be computed using the multiphysics coupling analysis software COMSOL. The magnetic diffusion equation and the Reynolds equation in the model are set by the software’s custom equations, and the iteration of the pressure of metal liquid film and film thickness can be computed by software COMSOL with MATLAB.

**Figure 6:**
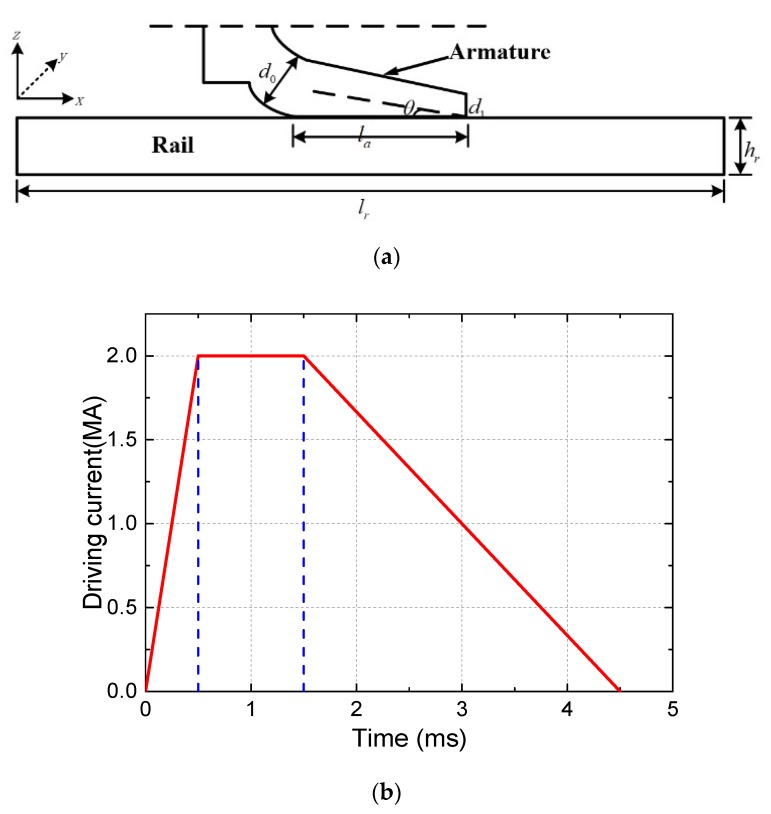
Geometry of A/R system and driving current: (**a**) two-dimensional schematic diagram of the A/R system, (**b**) driving current.

Figure 6a is a two-dimensional schematic diagram of the A/R system. The driving current of the A/R system is shown in Figure 6b.The armature is a C-shaped armature, and the structural parameters of the armature and rail are shown in Table 1.

The armature material is 7075 aluminum alloys and the rail material is copper alloys. Specific material parameters are shown in Table 2, and these data come from the COMSOL material library. The viscosity coefficient η of the metal liquid film in the table is related to the movement velocity of the liquid film. The calculation formula as follows:

(26) $$\eta =K_{T}\cdot \eta_{0}=(1+0.002166{\frac{\left(5\rho_{f}\cdot U\cdot roughness\right)}{\eta_{0}}}^{0.8265})\cdot \eta_{0}$$

### 3.2. Lubrication Characteristics of Metal Liquid Film at Specific Speed

**Figure 7:**
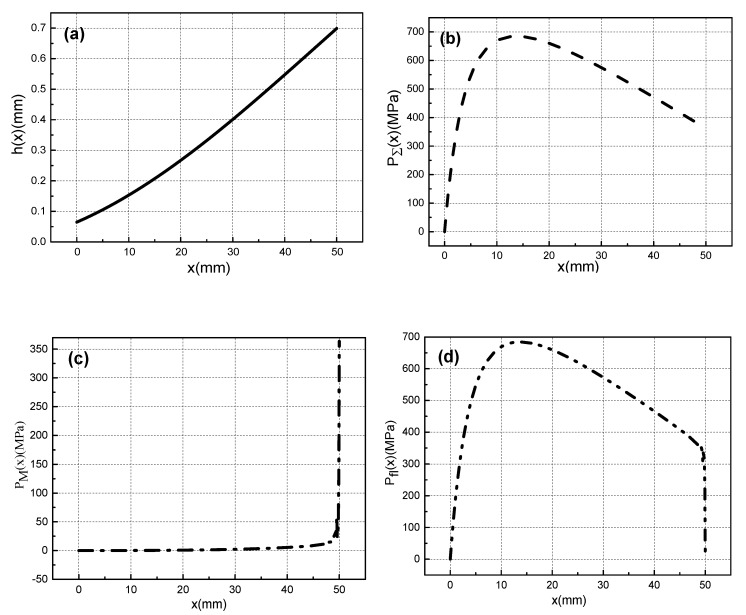
Lubrication characteristics of metal liquid film when the armature movement velocity is 1600 m/s: (**a**) Film thickness distribution, (**b**) Total pressure distribution, (**c**) Magnetic pressure curve, (**d**) fluid pressure.

According to the calculation method of Figure 4a, the pressure distribution and film thickness distribution of the metal liquid film at a certain speed can be obtained. Calculate according to the above simulation conditions, we can get the thickness curve of the metal liquid film h(x), the total pressure curve of the metal liquid film $P_{\Sigma }(x)$, magnetic pressure curve $P_{M}(x)$ and fluid pressure curve $P_{fl}(x)$ when the armature movement velocity is 1600 m/s, as shown in Figure 7a–d.

It can be seen from Figure 7a that the thickness of the metal liquid film is the thinnest at the armature head and the thickest at the end of the armature’s tail. Because of velocity skin effect, the current will be mainly concentrated on the end of the armature’s tail, and the electromagnetic force is the largest in the end of the armature. The armature is limited by the outer rail, which can only to deform inward, and the end of tail is more easily deformed than the head of tail. Therefore, the deformation of the armature’s tail end is larger than that of the head. Therefore, the metal liquid film will form a divergent gap distribution of the moving direction.

Under the combined effect of electromagnetic force and inertial force, the metal liquid film will enter the narrower head gap from the tail gap and then generate a great pressure, as shown in Figure 7b. This pressure can support the contact between the A/R, and ensure that the metal liquid film has a good lubrication effect. The total pressure of the metal liquid film include magnetic pressure and fluid pressure. Figure 7c is the magnetic pressure curve. When the armature moves at ultra-high velocity, the current is concentrated on the end of the armature’s tail (x = 50 mm) due to the velocity skin effect. Therefore, the magnetic pressure is at a maximum at this position. The fluid pressure in Figure 7d are caused by the fluid characteristics of the metal liquid film.

After calculating the total pressure distribution and thickness distribution of the metal liquid film, and then substituting it into the metal liquid film velocity calculation expression (6), the velocity distribution of the metal liquid film can be obtained. The velocity U(z) of the metal liquid film consists of two parts: one is the linear velocity distribution U_1_(z) caused by the lower surface rail motion in the metal liquid film, and the other is the parabola type velocity distribution U_2_(z) caused by the pressure gradient and electromagnetic force of the metal liquid film.

**Figure 8:**
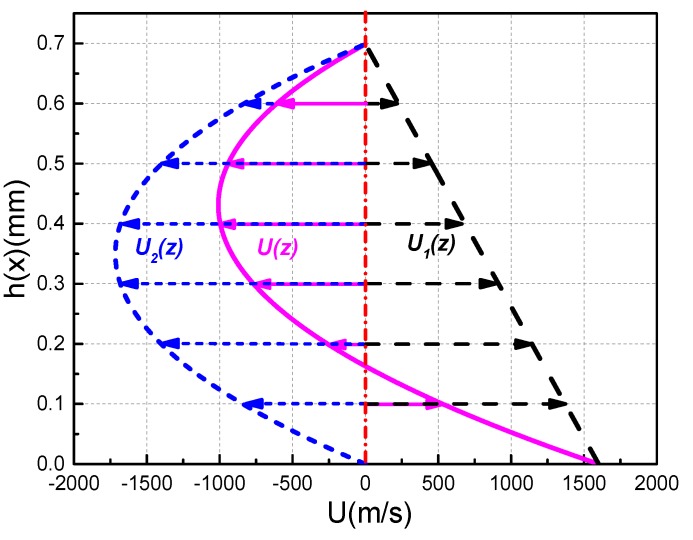
Velocity decomposition curves of metal liquid film at the exit of the contact surface at U = 1600 m/s.

The sign of U(z) is determined by the relative magnitude of U_1_(z) and U_2_(z). Figure 8 shows the curve of the liquid film velocity at the exit of the contact surface when the armature movement velocity is 1600 m/s. It can be seen from the Figure 8 that the velocity of the liquid film is positive near the side of rail, and gradually decreases as the height of the liquid film increases. When the absolute value of U_2_(z) is greater than U_1_(z), the velocity of the liquid film appears to be a negative value. At the same time, the liquid film velocity will gradually change from negative to zero because of the constraints of the boundary conditions. The presence of a negative value here simply means that the velocity direction of the liquid film is opposite to the reference direction.

**Figure 9:**
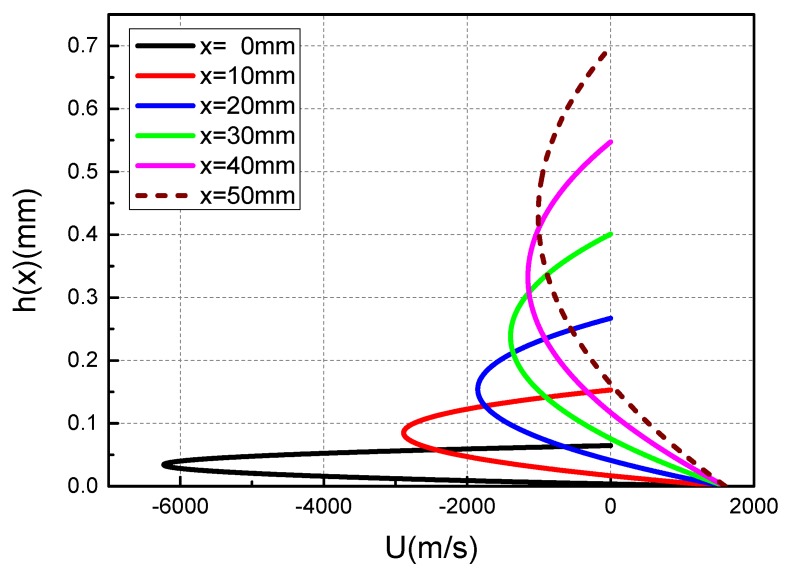
Velocity distribution curves of liquid film at different positions of the contact surface at U = 1600 m/s.

Using the metal liquid film velocity calculation expression, not only the velocity distribution of the metal liquid film at the contact surface exit can be calculated, but also the velocity distribution of the metal liquid film at different positions on the contact surface. Figure 9 shows the velocity distribution curves of the metal liquid film at different positions on the contact surface when the armature movement velocity is 1600 m/s.

**Figure 10:**
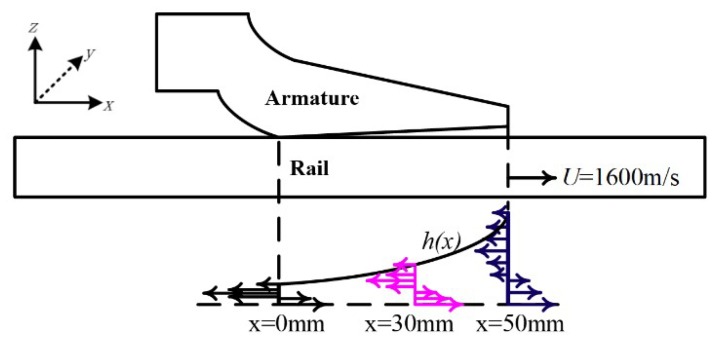
Velocity distribution schematic diagram of metal liquid film at different positions of the contact surface at U = 1600 m/s.

From the perspective of the overall distribution, the velocity distribution of different locations is similar to the velocity distribution of the exit, and there are negative values. At x = 0 mm (the head of armature’s tail), the absolute value of the negative value of the metal liquid film is the largest, reaching almost three times the velocity of armature movement. It can be seen that in areas where the metal liquid film is thinner, the velocity changes more drastically. Corresponding to the three velocity curves of x = 0, 30, and 50 mm in Figure 9, respectively, Figure 10 shows a schematic diagram of the velocity distribution of the metal liquid film at the contact surface at x = 0, 30, and 50 mm. The distribution of the metal liquid film velocity at different positions on the contact surface can be seen more intuitively from the Figure 10.

### 3.3. Lubrication Characteristics of Metal Liquid Film at Full Current Stage

**Figure 11:**
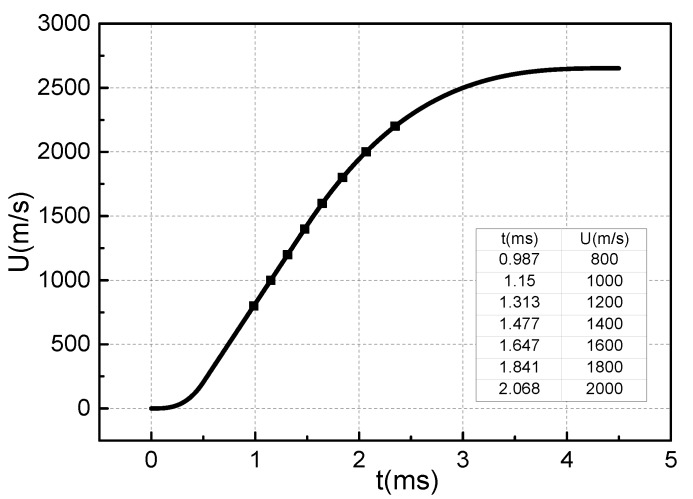
Armature velocity curve at full current stage.

**Figure 12:**
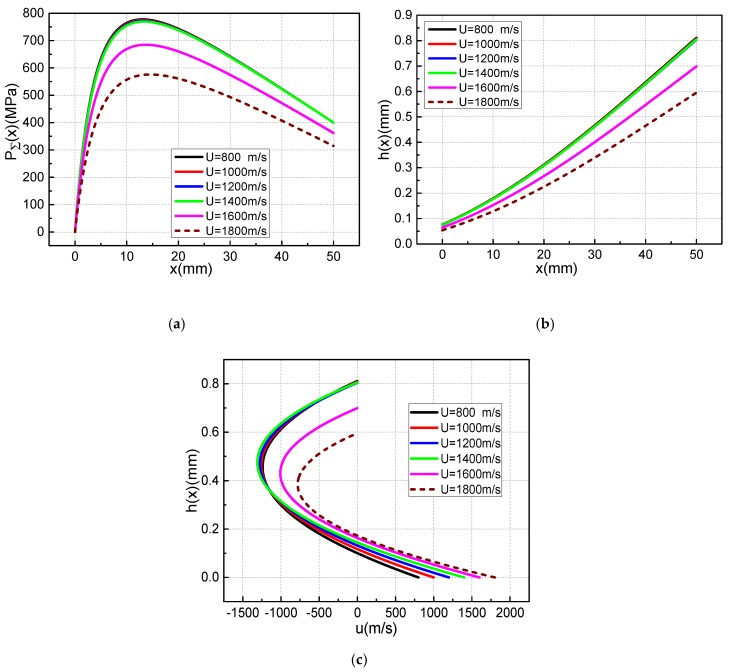
Distribution characteristics of total pressure, film thickness and velocity at the exit of metal liquid film at different armature velocity: (**a**) Total pressure distribution, (**b**) Film thickness distribution, (**c**) Velocity distribution at exit.

According to the driving current in Figure 6b, the distribution curve of the armature movement velocity can be obtained, as shown in Figure 11. Selecting armature velocity of 800, 1000, 1200, 1400, 1600, 1800, 2000, and 2200 m/s, and calculating according to the overall calculation flows in Figure 4b, we can obtain the lubrication characteristic distribution of the metal liquid film during the entire launch process, as shown in Figure 12.

The total pressure distribution and thickness distribution of the metal liquid film at different armature velocities in Figure 12 are similar to those in Figure 7. At the current flat-top phase, when the corresponding armature speed is 800–1400 m/s, the total pressure and thickness distribution of the metal liquid film are basically it remains unchanged. When the velocity of the armature is greater than 1400 m/s, the value of the maximum film thickness starts to decrease, and the maximum film thickness value decreases by about 12.5% for each 200 m/s increase in speed. The maximum total pressure was obtained at x = 12 mm, and the maximum total pressure decreased by approximately 11.3% for each 200 m/s increase in speed. The maximum velocity were obtained at 60% of the maximum film thickness. When the armature speed is less than 1400 m/s, the maximum velocity of the liquid film increases by 25% for each 200 m/s increase in speed.

**Figure 13:**
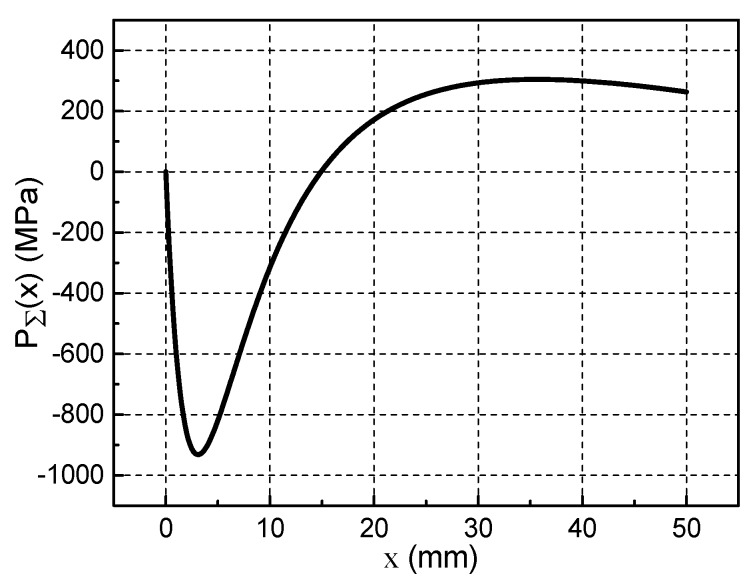
Total pressure of metal liquid film at U=2000 m/s.

**Figure 14:**
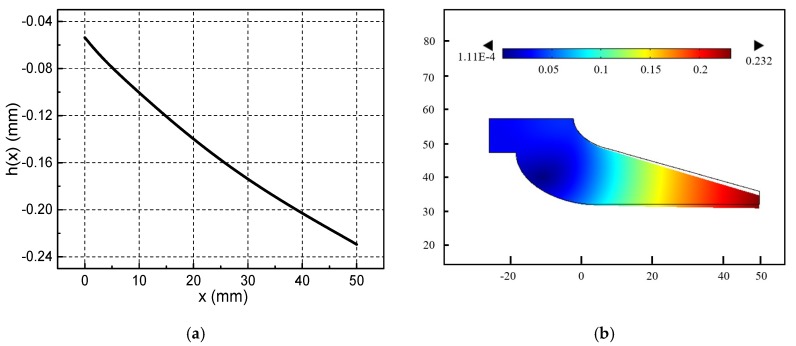
Thickness of metal liquid film at U = 2000 m/s:(**a**) Film thickness distribution of metal liquid film, (**b**) Deformation cloud diagram of armature’s tail.

The muzzle velocity of armature in Figure 11 can reach 2636.4 m/s, while Figure 12 only shows the distribution of the lubricating characteristics of the metal liquid film at an armature velocity of 800–1800 m/s. Figure 13 shows the pressure distribution curve obtained after several iterative calculations when the armature velocity is 2000 m/s. It can be seen that the total pressure of metal liquid film has a negative value. Figure 14 shows the thickness distribution curve of the metal liquid film and the cloud distribution of the armature deformation obtained from this calculation. It can be seen that the thickness of the liquid film has all become negative, and the armature’s tail is deformed to the rail side. This is the result of a change in the total pressure distribution of the metal liquid film. It can be seen from Figure 13 that the total pressure of the metal liquid film has a negative value of the armature’s head, and that its absolute value is much larger than the positive value of the end of the armature’s tail. This indicates that the total pressure direction of the metal liquid film is directed to the rail side. The end of the armature’s tail is more prone to deformation, therefore the maximum deformation occurs at this position, as shown in Figure 14b. The shoulder position of the armature’s tail has greater rigidity and the throat position is fixed. Therefore, the deformation of the position is small. Negative values of total pressure and film thickness indicate that it is impossible for the metal liquid film to exist between the armature and the rail, and the metal liquid film cannot function as a lubricant.

The friction force $F_{f}$ can be obtained by integrating the velocity gradient over the entire liquid film. At the same time, the current flowing in the metal liquid film will also be affected by the electromagnetic force $F_{em}$. The calculation expression is as follows:

(27) $$F_{f}=w_{a}\int_{0}^{l_{a}}{\eta \frac{\partial u}{\partial z}|}_{z=h}dx$$

(28) $$F_{em1}=\frac{1}{2}\cdot L’\cdot I^{2}$$

(29) $$F_{em2}=w_{a}{h(x)|}_{x=l_{a}}P_{M}$$

In the above formula, $F_{em1}$ represents the electromagnetic force received by the armature, and $F_{em2}$ represents the electromagnetic force received by the metal liquid film. L′ is the inductance gradient of the launch system, with a value of 0.45 μH/m, and I is the driving current waveform. The specific waveform is shown in Figure 6b.

**Figure 15:**
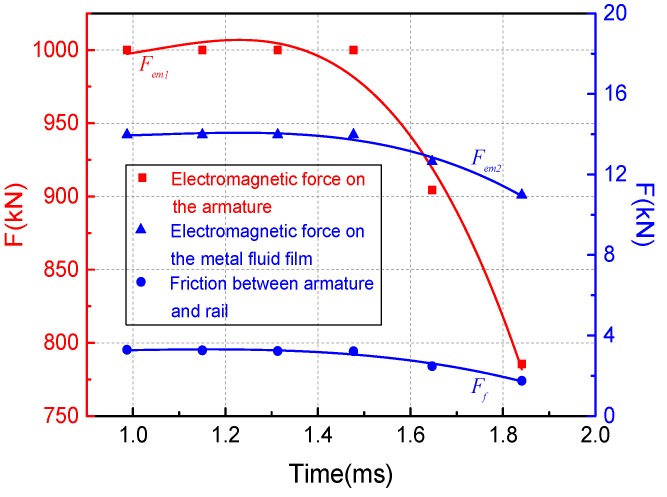
Electromagnetic force and frictional resistance during launch.

According to the above expressions, a scatter plot of frictional resistance and electromagnetic force over time can be calculated. On this basis, the data can be fitted to obtain the change law of friction resistance and electromagnetic during the entire movement process, as shown in Figure 15. It can be seen from Figure 15 that the average electromagnetic force experienced by the metal liquefaction layer at the full current stage is 13.25 kN, while the average friction force of A/R interface is only 2.87 kN. The electromagnetic force received by the liquid film is greater than the frictional resistance of rail and armature, and the electromagnetic force received by the armature is far greater than the frictional resistance. This also proves that the lubrication effect of the metal liquid film on the A/R contact interface is very significant.

## 4. Conclusions

In this paper, we describe in detail the process of deriving the Reynolds equation applicable to the metal liquid film, and consider the electromagnetic force that the metal liquid film may be subjected to. An electromagnetic-elastic mechanics-hydrodynamics multiphysics coupling model was established to study the lubrication characteristics of metal liquid film. Using this model, we first analyzed the lubrication characteristics of the metal liquid film at an armature speed of 1600 m/s, and obtained the hydraulic pressure distribution, film thickness distribution, and liquid film velocity distribution. The calculation results show that the maximum value of total pressure of the metal liquid film reached 681.86 MPa, and the maximum value of film thickness reached 0.7 mm. It was also found that the closer to the armature’s head, the higher the velocity of the liquid film. The maximum velocity of the liquid film at the armature’s head position was three times that of the end position. The hydraulic pressure is a divergent gap between the direction of the armature movement, however, because of the velocity skin effect, the current flows from the end of the armature’s tail to the armature body, and a large magnetic pressure is formed here, the maximum value reached 370 Mpa. The large magnetic pressure can make up the original divergent gap and makes the liquid film between the rail and armature can exist stably and has a good lubrication effect.

Furthermore, we analyzed the lubrication characteristics of the metal liquid film at the full current stage. The study found that in the flat phase of the current, the total hydraulic pressure distribution, velocity distribution, and film thickness distribution of the metal liquid film were basically the same. When the velocity of the armature is greater than 1400 m/s, the value of the maximum film thickness starts to decrease, and the maximum film thickness value decreases by about 12.5% for each 200 m/s increase in speed. The maximum total pressure was obtained at x = 12 mm, and the maximum total pressure decreased by approximately 11.3% for each 200 m/s increase in speed. The maximum velocity were obtained at 60% of the maximum film thickness. When the armature speed is less than 1400 m/s, the maximum velocity of the liquid film increases by 25% for each 200m/s increase in speed. At the same time, when the armature movement speed is too large, the hydraulic pressure and film thickness have negative values. It means that the metal liquid film cannot exist stably at this time, and the liquid film may break, which may be one reason for transition that often occurs to the falling phase. It is an important research direction to extend the life of railgun barrel to understand the mechanism of transition caused by the rupture of the liquefaction layer and avoid this transition.

Finally, this paper analyzes the lubricating effect of the liquid film during the entire launch phase. The results show that the average electromagnetic force experienced by the metal liquefaction layer at the full current stage is 13.25 kN, while the average friction force of A/R interface is only 2.87 kN. This means that the appearance of the metal liquid film has a good lubricating effect on the contact interface of the A/R. One of the major requirements of electromagnetic railguns is controllable speed. Therefore, only by fully understanding the lubricating effect of the metal liquid film on the armature and the rail can it ensured that the muzzle velocity of the projectile is effectively controlled during the launch process.

The influence of the armature geometry and the material parameters of the armature and rail on the lubrication effect of the metal liquid film needs further research.

## Figures and Tables

**Table 1:** Structural parameters of armature and rail.

$\mathrm{Length}\;\mathrm{of}\;\mathrm{Tail},\;l_{a}$	$\mathrm{Thickness}\;\mathrm{of}\;\mathrm{Tail}’s\;\mathrm{End},\;d_{1}$	Tail Angle, θ
50 mm	4.75 mm	12.95°
Thickness of throat, $d_{0}$	Length of rail, $l_{r}$	Width of rail, $h_{r}$
22.22 mm	200 mm	15 mm

**Table 2:** Material properties.

**Rail Material Properties**	
Density, $\rho_{r}$	8700 kg/m^3^
Conductivity, $\sigma_{r}$	5.998 × 10^7^ S/m
Thermal conductivity, $k_{r}$	400 W/(m·K)
Specific heat capacity, $c_{r}$	385 J/(kg·K)
**Armature Material Properties**	
Density, $\rho_{a}$	2700 kg/m^3^
Conductivity, $\sigma_{a}$	3.774 × 10^7^ S/m
Thermal conductivity, $k_{a}$	238 W/(m·K)
Specific heat capacity, $c_{a}$	900 J/(kg·K)
Melting temperature, $T_{m}$	805 K
Latent Heat, *H*	3.78 × 10^5^ J/kg
Young’s modulus, *E*	7.0 × 10^10^ Pa
Poisson’s ratio, μ	0.33
**Metal Liquid Film Material Properties**	
Density, $\rho_{f}$	2485 kg/m^3^
Conductivity, $\sigma_{f}$	4.5 × 10^6^ S/m
Thermal conductivity, $k_{f}$	90.97 W/(m·K)
Specific heat capacity, $c_{f}$	1084 J/(kg·K)
Initial value of viscosity coefficient, $\eta_{0}$	0.0045 Pa·s
viscosity coefficient, η	$\eta_{0}\cdot K_{T}$
roughness	1.42 μm
